# The online promotion strategies of e-cigarette and heated tobacco product retailers in South Korea following the COVID-19 pandemic: Implications for regulation

**DOI:** 10.18332/tid/178380

**Published:** 2024-02-14

**Authors:** HyoRim Ju, HyeWon Lee, Juyoung Choi, Soojeong Kim, EunKyo Kang

**Affiliations:** 1 Department of Family Medicine, Dankook University Hospital, Cheonan, South Korea; 2 Department of Family Medicine, Seoul National University College of Medicine, Seoul, South Korea; 3 National Cancer Control Institute, National Cancer Center, Goyang, South Korea; 4 Department of Biomedical Sciences, Seoul National University, Seoul, South Korea; 5 Department of Family Medicine, National Cancer Center, Goyang, South Korea

**Keywords:** e-cigarettes, heated tobacco products, promotion, online promotion, retailers

## Abstract

**INTRODUCTION:**

The surge in popularity of e-cigarettes and heated tobacco products (HTPs) in South Korea, driven by perceived health benefits and COVID-19-related concerns, has led to increased advertising claims about their safety despite ongoing debates about their health effects. This study explores the marketing strategies of online e-cigarette and HTP retailers in South Korea pre- and post-COVID-19, examining potential misleading claims and providing a foundation for future regulatory measures.

**METHODS:**

We conducted a comprehensive study of eight major e-commerce platforms and three dominant search engines in South Korea to analyze the marketing and advertising strategies of e-cigarettes and heated tobacco products (HTPs) (n=774). Using specific keywords, promotional strategies were identified and categorized, after which statistical analysis was conducted to understand the frequency and proportion of these strategies, highlighting differences between HTP and e-cigarette sellers.

**RESULTS:**

Our analysis reveals a significant rise in the number of online retailers selling e-cigarettes and HTPs following the COVID-19 pandemic, with the promotional strategies ‘Stay home and vape’ and ‘Trendy’ being the most prevalent. Trends also indicate a shift in promotional strategies over the years, with a marked increase in health reassurance themes and appeals to trendiness, particularly targeting female consumers, which were used significantly more at HTPs stores.

**CONCLUSIONS:**

The study highlights the need for stricter regulation due to the potential health risks posed by the aggressive marketing strategies of e-cigarette and HTP online retailers in South Korea, amplified by the COVID-19 pandemic.

## INTRODUCTION

Since the introduction of heated tobacco products (HTPs) in the late 2000s, various advertisements have been promoting e-cigarettes and HTPs, claiming that e-cigarettes and HTPs are less harmful *smoking* alternatives to traditional cigarettes^1,2^. This perception has increased the interest in e-cigarettes and HTPs, which has been boosted by the heightened concern about the increased risk of infection and severity of the coronavirus disease 2019 (COVID-19) for people who smoke cigarettes, and the rise in anxiety about the health impacts of traditional smoking^3^. As a result, messages of health benefit claims in e-cigarette and HTPs advertisements and promotions have increased. In South Korea, especially after the COVID-19 pandemic, there has been a high preference for HTPs, and the sales volume of HTPs in 2021 reached 210 million packs, an increase of 16.3% compared to the previous year. In addition, HTPs sales volume is increasing every year, in particular among smokers, and the preference for e-cigarettes and HTPs is high among young people^4^.

However, the health effects of e-cigarettes and HTPs are still a topic of debate. While some studies suggest that e-cigarettes and HTPs reduce the risk of lung diseases, such as cancer and chronic obstructive pulmonary disease (COPD), compared to traditional cigarettes^5,6^, others suggest that HTPs increase respiratory symptoms and impair lung function^7,8^ and contain similar or higher levels of certain carcinogens, such as nicotine, tar, formaldehyde, and benzene, compared to traditional cigarettes^9,10^. The World Health Organization (WHO) has adopted a decision at the 8th Conference of the Parties to the Framework Convention on Tobacco Control (FCTC) that calls for applying the same policies and regulations to e-cigarettes and HTPs, acknowledging the risks associated with these products^11^. As a member country of the FCTC, South Korea has strengthened regulations on e-cigarettes and HTPs in line with the increasing obligation to comply with the treaty and the growing preference for e-cigarettes among young people, along with a continuous increase in the sales volume of e-cigarettes and HTPs^12^.

However, South Korea’s tobacco-related laws have a narrow definition of tobacco products, which means that various e-cigarettes and HTPs are not included^13^. Despite the ban on online sales and advertising for traditional cigarettes, e-cigarettes and HTPs are still sold online. Parallel to the increased sales of e-cigarettes and HTPs since the COVID-19 pandemic began, advertisements promoting the health benefits of e-cigarettes and HTPs as an alternative to traditional cigarettes have also increased^14,15^.

For this reason, this study examines the marketing and advertising strategies of South Korea-based online e-cigarette and HTP retailers since the domestic COVID-19 pandemic began. We analyze the current status of advertisements or promotional strategies for e-cigarettes and HTPs that may mislead the public about the effects of these products. We also provide a basis for effective regulation and monitoring measures in the future. This includes the need for effective regulatory measures and monitoring for e-cigarettes and HTPs due to the potential for incorrect perceptions caused by advertising or promotion.

## METHODS

### Search procedure

We collected data from the top eight e-commerce platforms in South Korea, ranked by Korean e-commerce rankings^16^. The platforms included Naver Shopping, Coupang, eBay South Korea, 11st, Lotte On, SSG.com, WeMakePrice, and TMON. These platforms accounted for two-thirds of the total platform market share^16^. To collect webpages that sell e-cigarette products independently, we also conducted searches on Naver, Google South Korea, and Daum. In South Korea, the search platforms Naver, Daum, and Google South Korea dominate the market as of 2022, with Naver holding over 70% of the market share within South Korea^16^.

In January 2023, we searched the following keywords to collect data: ‘e-cigarette (jeonjadambae)’, ‘e-cig (jeondam)’, ‘e-liquid (aeksang)’, ‘cigarlike (yusadambae)’, ‘e-liquid cigarette (aeksangdambae)’, ‘heated tobacco product (gwollyeonhyeong jeonjadambae)’, ‘HTP (gwollyeon jeondam)’, and ‘cigarette (dambae)’. To ensure a comprehensive search process, we also separately searched for the names of high-market share e-cigarette and HTP brands as of 2022, including: IQOS, Glo, Lil, JUUL, Aspire, and Justfog. When searching for the top five HTP or e-cigarette product names with high sales volume, we confirmed that all online retailer sites found were also included in the search results of the tobacco-related keywords. Therefore, we confirmed that our keyword searches did not miss any retailer online sites. Our search was conducted in January 2023 and included data from website pages uploaded before January 2023.

To identify duplicate web pages, the URLs of the collected online retail website pages were compared and checked for duplicates. If the URLs were different, but the seller’s name and product name were identical, we assumed that the same retailer was selling on multiple platforms and removed the duplicate website pages from the dataset. Data on the price of the HTPs and e-cigarettes being sold, offering free shipping, and the start date of sales (the date the website page was opened) were collected. In this study, the name and mechanism of products on sale were identified on each page, and each product was classified into HTP and e-cigarette. Pages selling tobacco-like products that do not fall under either category were removed from the data list. As a result, the website pages of a total of 774 online retailers were analyzed.

### The definitions of promotional strategies

The definitions established by previous studies to define the content of promotional strategies were applied^17^. The definitions were categorized into seven groups.

*Stay home and vape*

This was defined as messaging promoting the ability to purchase products online, the availability of phone ordering with delivery, or contactless delivery during the COVID-19 pandemic. It also includes cases where retailers claim their product does not produce cigarette-like odors and could be used for indoor smoking since indoor smoking often causes disputes between neighbors in Korea.

*Trendy (trendiness)*

This was defined as messaging that promotes the use of the products as stylish and trendy; as a promotional strategy that targets women and promotes the products as clean and stylish for use by women or advertises the products as more sophisticated compared to cigarettes. It also conveys messages that the products increase social acceptability or are related to the trendsetter or looking ‘cool’, ‘young’, and ‘stylish’ image.

*Health reassurance themes*

These were identified as promoting the products as relatively less harmful to health. For example, including claims that their product emits fewer harmful substances, the principle that the product itself is less harmful, or the product could be used as an alternative to smoking cessation.

*Buy our vaping product, receive a free gift of essential supplies*

This promotional strategy offers various products, such as e-cigarette devices, cleaning devices, masks, or hand sanitizers, as a gift for purchasing the vaping product. Some retailer sites also offered free gifts through a lucky draw or provided free gifts for writing good reviews.

*COVID-19-themed discounts*

This refers to discounts based on pandemic-protective measures^17^. This includes discounts for healthcare workers as well as promotions that offer discounts during the COVID-19 epidemic to allow customers to continue receiving nicotine products without leaving their homes.

*Vaping will help you to cope with the COVID-19 pandemic*

This is defined as a promotional strategy used by online retailers that claim their products can help alleviate stress and anxiety or provide supposed benefits for coping with the COVID-19 pandemic.

*COVID-19 safety assurance themes*

This is when e-cigarette brands express safety messages regarding their product manufacturing and handling in response to fears that customers may avoid their products due to concerns about COVID-19 infection. This definition includes promoting the daily sanitation of workspaces, wearing protective equipment while making the products, and other related safety measures as part of their promotional strategy^17^.

### Data coding and analysis

According to the above definitions, two coders labeled the data independently; the first coder completed the coding, and then the second independent coder performed the same procedure separately. This method is commonly used in data labeling tasks to verify consistency between coders and minimize labeling errors^18^. If the result of the two coders matched, that labeling was confirmed. If the coding of the two coders did not match, a third independent coder performed the coding process, and the coding agreed upon by two of the three coders was confirmed as the retailer’s promotion strategy.

To determine the inter-rater reliability, 10% of the entire dataset was randomly sampled and coded by a third independent researcher. The reliability was calculated by Cohen’s kappa and showed a result of 0.95 (95% CI: 0.92–0.98, p<0.001). To consider the possibility of human error, a text-data classification model was developed and applied to evaluate the reliability. After extracting text from images on the online retailers’ website pages, we preprocessed the text using Python’s KoNLPy package^19^. The data were tokenized, and punctuation, stop words, and special characters were removed. Then, we performed morphological analysis and lemmatization. Afterwards, the accuracy of the text classification model was evaluated. To create the data classification model, 20% of the data labeled by the two coders were randomly sampled, and another 20% were randomly sampled for model evaluation. The accuracy of the model was 92.3%, and the reliability of the first and second coders was 0.93 and 0.92, respectively, as evaluated by applying the model to the labeled dataset.

To perform data analysis, the labeled data were used to calculate the frequency and proportion of the inclusion of promotional strategies. Additionally, Pearson’s chi-squared test was performed to analyze the difference in the frequency of the use of promotional strategies by year and the difference between HTP sellers and e-cigarette sellers. The text preprocessing procedures for developing a classification model and statistical analysis were performed using Python (version 3.11.1) and R (version 4.0.4). For all analyses, a 95% CI that excluded unity, and a p<0.05, was considered evidence of statistical significance and all tests were two-tailed. Analyses and results were done and visualized using R (version 4.0.4). It was not appropriate or possible to involve patients or the public in the design, conduct, reporting, or dissemination plans of our research.

## RESULTS

**Figure 1:**
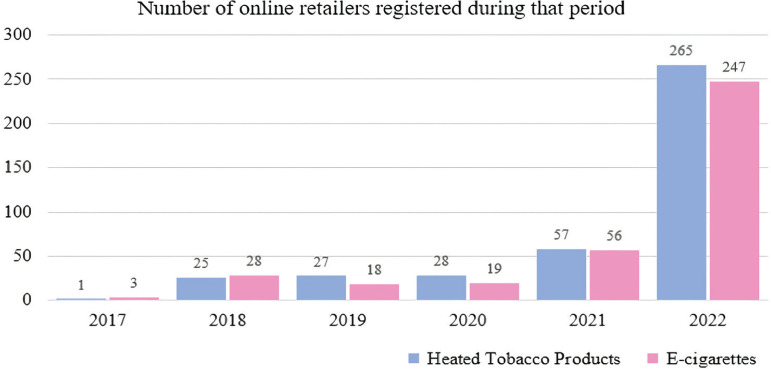
Number of online e-cigarette and HTP retailers registered in South Korea following the COVID-19 pandemic

Table 1 displays the data on the number of online websites selling e-cigarette and HTPs, average price, and the number of online retailers offering free shipping. The registration date of the online e-cigarette and HTP websites varied from 2017 to 2022. The largest proportion of websites was registered in 2022, accounting for 66.2%, followed by 2021. It shows that after the COVID-19 pandemic began, there was a significant increase in the number of online retailers offering e-cigarettes and HTPs. In 2017, there were four online retailers (0.5% of the total) with an average price of US$ 76.3 ± 7.2, and one retailer (25.0%) offered free shipping. In 2018, there were 53 retailers (6.9%) with an average price of US$ 72.0 ± 30.7, and 26 retailers (49.1%) provided free shipping. In 2019, 45 online retailers (5.8%) were observed, with an average price of US$ 70.0 ± 29.6, and 20 retailers (44.4%) offered free shipping. In 2020, there were 47 online retailers (6.1%) with an average price of US$ 58.3 ± 15.8, and 29 retailers (61.7%) provided free shipping. In the following year, 2021, there were 113 retail websites (14.6%) with an average price of US$ 52.5 ± 14.8, and 63 retailers (55.8%) offered free shipping. Lastly, in 2022, 512 online retailers (66.2%) were recorded, with an average price of US$ 63.9 ± 27.8, and 304 retailers (59.4%) provided free shipping. Since 2021, when the COVID-19 pandemic peaked, the number of online retailers has shown an increasing trend (Figure 1). In particular, both e-cigarettes and HTPs showed an increasing trend, and in 2022, a markedly increasing trend was observed.

**Table 1:** Numbers, average price and number of e-cigarette and HTP retailers offering free shipping in South Korea following the COVID-19 pandemic

*Year*	*n (%)*	*Price (US$) Mean ± SD*	*Free shipping*
2017	4 (0.5)	76.3 ± 7.2	1 (25.0)
2018	53 (6.9)	72.0 ± 30.7	26 (49.1)
2019	45 (5.8)	70.0 ± 29.6	20 (44.4)
2020	47 (6.1)	58.3 ± 15.8	29 (61.7)
2021	113 (14.6)	52.5 ± 14.8	63 (55.8)
2022	512 (66.2)	63.9 ± 27.8	304 (59.4)
**Total**	774 (100)	62.9 ± 26.4	443 (57.2)

Table 2 shows the seven promotional strategies for e-cigarettes and HTPs. Most retailers (57.5%, n=445) used the promotional strategy ‘*Stay home and vape*’. This strategy was applied significantly more by HTP sellers than by e-cigarette products (61.8% vs 52.8%, p=0.012). The second most popular promotional strategy (44.4%, n=344) was ‘*Trendy*’. Promotion strategies emphasizing trendiness were also used significantly more by HTP sellers than e-cigarette products (49.9% vs 38.5%, p=0.012). The third and fourth most popular promotional strategies used by the retailers were ‘*Buy our vaping product, receive a free gift of essential supplies*’ (24.2%, n=187) and ‘*COVID-19 themed discounts*’ (15.5%, n=104), respectively. The other five strategies, including the above two strategies, did not show a significant difference between HTP sellers and e-cigarette sellers.

**Table 2:** Promotional strategies of e-cigarette and HTP retailers in South Korea following the COVID-19 pandemic

*Strategies*	*Total n (%)*	*Heated tobacco products n (%)*	*E-cigarettes n (%)*	*p*
Stay home and vape	445 (57.5)	249 (61.8)	196 (52.8)	0.012
Trendy	344 (44.4)	201 (49.9)	143 (38.5)	0.002
Health reassurance themes	115 (14.9)	56 (13.9)	59 (15.9)	N/S
Buy our vaping product, receive a free gift of essential supplies	187 (24.2)	96 (23.8)	91 (24.5)	N/S
COVID-19 themed discounts*	104 (15.5)	63 (15.6)	41 (11.0)	N/S
Vaping will help you to cope with the COVID-19 pandemic*	3 (0.5)	3 (0.7)	0 (0.0)	-
Our products will not give you COVID-19*	4 (0.6)	2 (0.5)	2 (0.5)	N/S
**Total**	774 (100)	403 (52.1)	371 (47.9)	

* Since the COVID-19 outbreak arose after 2020, only online sellers registered in 2020–2022 were analyzed.

Table 3 presents the promotional strategies used by online e-cigarette and HTP retailers before 2020 to 2022. The proportion of retail websites that applied the promotional strategy ‘*Stay home and vape*’ increased from 40.2% (before 2020) to 57.5% in 2020, 55.8% in 2021, and 61.3% in 2022 (p<0.001). In the case of HTP online stores, it was confirmed that the number of stores using this promotional strategy increased significantly according to year (p=0.025). The promotional strategy, ‘*Trendy*’, was used by 34.3% of retailers’ websites before 2020 and increased to 55.3% in 2020, 52.2% in 2021, and 43.8% in 2022 (p=0.025). The strategies, including ‘*COVID-19 themed discounts*’, were used by 34.0% of the retailers in 2020; however, by 2022, this number decreased significantly to 8.2% (p<0.001). The number of retailers using this strategy decreased significantly from 2020 to 2022 on both HTP and e-cigarette retailers’ websites.

**Table 3:** Promotional strategies of e-cigarette and heated tobacco products (HTPs) retailers in South Korea by year following the COVID-19 pandemic

*Strategies*	*Product*	*Year*				*p*
		*Before 2020 n (%)*	*2020 n (%)*	*2021 n (%)*	*2022 n (%)*	
**Stay home and vape**	E-cigarettes	16 (32.7)	11 (57.9)	30 (53.6)	139 (56.3)	N/S
	HTPs	25 (47.2)	16 (57.1)	33 (57.9)	175 (66.0)	0.025
	Total	41 (40.2)	27 (57.5)	63 (55.8)	314 (61.3)	0.001
**Trendy**	E-cigarettes	13 (26.5)	8 (42.1)	26 (46.4)	96 (38.9)	N/S
	HTPs	22 (41.5)	18 (64.3)	33 (57.9)	128 (48.3)	0.045
	Total	35 (34.3)	26 (55.3)	59 (52.2)	224 (43.8)	0.025
**Health reassurance themes**	E-cigarettes	10 (20.4)	3 (15.8)	7 (12.5)	39 (15.8)	N/S
	HTPs	10 (18.9)	8 (28.6)	6 (10.5)	32 (12.1)	N/S
	Total	20 (19.6)	11 (23.4)	13 (11.5)	71 (13.9)	N/S
**Buy our vaping product, receive a free gift of essential supplies**	E-cigarettes	11 (22.5)	4 (21.1)	15 (26.8)	61 (24.7)	N/S
	HTPs	7 (13.2)	8 (28.6)	19 (33.3)	62 (23.4)	N/S
	Total	18 (17.7)	12 (25.5)	34 (30.1)	123 (24.0)	N/S
**COVID-19 themed discounts**	E-cigarettes		5 (26.3)	17 (30.4)	19 (7.7)	<0.001
	HTPs		11 (39.3)	29 (50.9)	23 (8.7)	<0.001
	Total		16 (34.0)	46 (40.7)	42 (8.2)	<0.001
**Vaping will help you to cope with the COVID-19 pandemic**	E-cigarettes		0 (0.0)	0 (0.0)	0 (0.0)	-
	HTPs		1 (3.6)	0 (0.0)	2 (0.8)	N/S
	Total		1 (2.1)	0 (0.0)	2 (0.4)	N/S
**Our products will not give you COVID-19**	E-cigarettes		0 (0.0)	0 (0.0)	2 (0.8)	N/S
	HTPs		0 (0.0)	1 (1.8)	1 (0.4)	N/S
	Total		0 (0.0)	1 (0.9)	3 (0.6)	N/S

Table 4 presents the detailed contents of two major promotional themes found on the online e-cigarette and HTP retail websites: ‘*Health reassurance themes*’ and ‘*Trendy*’. Regarding the details of the ‘*Trendy*’ promotional strategy, the greatest emphasis was placed on marketing strategies appealing to female smokers (35.2%). The promotional strategy to appeal to female smokers as trendy was used significantly more at e-cigarette stores; specifically, messages included claims that their products were suitable for female smokers because they produced no odor and that women who use e-cigarettes are sophisticated. The next most common detailed content was ‘Using this product is catching up with the fad’ at 29.7%, followed by ‘Using this product is cool’ at 18.0%. The above two strategies were used more by HTP online sellers than e-cigarette sellers. Regarding ‘*Health reassurance themes*’, the majority of retailers incorporated the promotional strategy ‘Harmful substances are released less’ (85.2%). This promotional strategy was used significantly more at HTP stores. The next most prevalent content was ‘Principle of this product is less harmful’ (38.2%), followed by ‘An alternative to smoking cessation’ (24.3%).

**Table 4:** Detailed contents of two major online promotional strategies of e-cigarette and HTP retailers in South Korea following the COVID-19 pandemic

*Strategies*	*Detail contents*	*Total n (%)*	*Heated tobacco products n (%)*	*E-cigarettes n (%)*	*p*
**Trendy**	Appeal to female smokers	121 (35.2)	42 (20.9)	79 (55.2)	<0.001
	Sophisticated compared to cigarettes	45 (13.1)	37 (18.4)	8 (5.6)	
	Using this product is catching up with the fad	102 (29.7)	63 (31.3)	39 (27.3)	
	Using this product is cool	62 (18.0)	42 (20.9)	20 (14.0)	
	Other	38 (11.0)	20 (10.0)	18 (12.6)	
**Health reassurance themes**	Harmful substances are released less	98 (85.2)	52 (92.9)	46 (78.0)	<0.001
	Principle of this product is that it is less harmful	44 (38.3)	31 (55.4)	13 (22.0)	
	An alternative to smoking cessation	28 (24.3)	9 (16.1)	13 (32.2)	
	Other	13 (11.3)	5 (8.9)	19 (13.6)	

## DISCUSSION

This study analyzed the websites of online e-cigarette and HTP retailers based in South Korea, established between 2017 and 2022. The results revealed a significant increase in the number of online retailers, particularly in 2022, following the outbreak of the COVID-19 pandemic. The most frequently used promotional strategies were ‘*Stay home and vape*’ and ‘*Trendy*,’ which showed an upward trend from 2020 to 2022. This trend was particularly evident in HTPs compared to e-cigarettes. The ‘*Stay home and vape*’ strategy emphasized the convenience of online shopping without the need to go out and the minimal impact on the surrounding environment due to the absence of odor when using indoors. However, the potential harm of indirect vaping from e-cigarettes and HTPs remains largely unknown^20-22^. And ‘*COVID-19 themed discounts*’ were popular during the COVID-19 pandemic but decreased significantly by 2022. Additionally, the ‘*Health reassurance themes*’ and ‘*Trendy*’ promotions emphasized the appeal of e-cigarettes and HTPs products to female smokers, claimed reduced harmful substances, and provided alternative smoking cessation options.

According to previous studies, e-cigarettes and HTPs marketing, health-related benefits, and low prices have been found to be the most commonly used promotional strategies^23^. However, in our study, we found that ‘*Stay home and vape*’ was the most commonly used promotional strategy among the e-cigarette and HTP retail websites. Although the health reassurance themes mentioned in previous studies are still employed as advertising strategies, ‘*Stay home and vape*’ consistently increased during the COVID-19 period^24^. This is a result of emphasizing the ease of purchasing and using e-cigarettes or HTPs at home during isolation caused by COVID-19. This result could ultimately increase the number of e-cigarette and HTP users compared to traditional cigarette products^25^. In addition, marketing that emphasizes the use of e-cigarettes and HTPs ‘anywhere’, including indoor spaces where smoking is restricted, could induce smokers to use e-cigarettes or HTPs and potentially increase nicotine consumption. Furthermore, the indoor use of e-cigarette devices can expose users to unknown toxins. Volatile organic compounds, nicotine, and tobacco-related carcinogens have been found to exist in e-cigarette and HTP vapor, and heavy metals not present in cigarette smoke have also been found in e-cigarette vapor^20,21,26,27^.

Furthermore, as shown in our study, promoting e-cigarette use by emphasizing its ‘trendiness’ could lead to the glamorization of smoking through e-cigarette device use^28^. Traditional tobacco companies actively used celebrity endorsements to glamorize smoking. Such an emphasis on ‘trendiness’ has been reported to be associated with adolescent and female smoking, which has a positive reputation among adolescents and can also influence their smoking initiation^29^. Moreover, this promotional strategy can reduce negative beliefs or concerns about nicotine addiction by conveying that e-cigarette products or HTPs are less risky and more socially attractive than traditional cigarettes, which can encourage smoking initiation among young non-smokers and female non-smokers^30-32^.

In this study, we also found that many e-cigarette and HTP retailers still use promotional strategies that emphasize the health benefits of their products. The use of health promotion strategies based on consumers’ perceptions has also been noted in previous studies^33-35^; according to research on e-cigarette users recruited online, e-cigarettes are perceived to be less toxic compared to traditional cigarettes, and are recognized as smoking cessation devices^23,34,36^. However, little is known about the long-term health risks of e-cigarette or HTP use. E-cigarette retailers may also use promotional strategies that suggest using e-cigarette devices for smoking cessation, but claims of superiority in smoking cessation have not been proven^15,37^. Previous research suggests that e-cigarette use would have minimal effects on smoking status^38,39^. Even among smokers who reported using e-cigarettes to quit smoking traditional cigarettes, there was no difference in the quit rate between e-cigarette users and non-users. Therefore, e-cigarette products should be regulated as tobacco products in South Korea, and health and smoking cessation claims made by online e-cigarette and HTP retail websites should be monitored. Such promotional strategies can potentially create false perceptions among current smokers and future smoking populations, and should be monitored as one of the promotion strategies that require caution.

The marketing strategies of e-cigarette and HTP manufacturers and retailers are rapidly evolving and expanding^32,40,41^. However, the regulatory framework often lags behind consumer behavior, and restrictions that affect the online purchase of e-cigarettes and HTPs are only discussed after the number of users has increased^42^. In South Korea, e-cigarette devices and heated tobacco products are classified as general consumer goods, not tobacco products. Therefore, they are not obliged to include health warnings or comply with tobacco-related regulations^42^. The availability of free shipping and online purchasing, along with the introduction of stylish e-cigarette devices and the promotion of their health benefits and trendiness, may create the perception that e-cigarettes and HTPs are not harmful tobacco products but trendy and popular items. Tobacco companies also utilize these tactics to advertise and promote their products aggressively. However, as e-cigarettes and heated tobacco products are currently classified as general consumer goods, there are no appropriate regulatory measures to control improper promotional strategies.

Based on our results, there is a need to pay more attention to the marketing strategies of e-cigarette and HTP retailers. Additionally, to draw attention to the harmful effects of HTPs and promote them more strategically, many online retailers of HTPs are promoting them by mentioning that using HTPs is possible indoors and that they are trendy. This was a more noticeable characteristic among HTPs sellers compared to e-cigarette sellers. However, considering the health hazards of HTPs, such indiscriminate online promotion may increase the number of HTPs users and harm public health. In particular, because most HTPs are sold by tobacco companies, there is a tremendous potential for expansion in marketing budget, scope, and sophistication. Given the potential for e-cigarette and HTP promotion strategies to lead to smoking initiation and increased smoking prevalence, e-cigarette and HTP promotions among retailers should, like tobacco advertising, especially HTPs, be banned worldwide.

### Limitations

This study has several limitations. First, we searched for online retail sites in South Korea. Second, we could not find all e-cigarette and HTP retail websites on the internet. As the e-cigarette product market is rapidly changing and the associated websites are frequently changing, our analysis may not be generalizable to current retail websites. However, we focused on developing a methodology for searching for results that potential e-cigarette and HTP consumers are most likely to encounter. Furthermore, South Korea is one of the countries with the most advanced online market platforms, and given the explosive growth in the use of HTPs and e-cigarettes, this study is sufficient to understand the risks of promotional strategies. Lastly, due to limitations in the statistical analysis of the non-parametric chi-squared test, we were unable to account for other socio-political factors influencing smoking beyond the online promotion strategies.

## CONCLUSIONS

The aggressive promotional strategies employed by e-cigarette and HTP online retailers are a result of inadequate regulations. These promotions have intensified, particularly after the onset of the COVID-19 pandemic, with increased emphasis on online purchases, free shipping, and trendy advertisements targeting women and young individuals. However, the current situation in South Korea lacks regulations specifically addressing e-cigarettes and HTPs. To address the resulting harm, it is crucial to regulate e-cigarettes and HTPs under the category of ‘tobacco’. While some countries have already implemented tobacco-like regulations for e-cigarettes and HTPs, the majority of countries still lack proper regulations^43^. The World Health Organization Framework Convention on Tobacco Control (WHO FCTC) and its member countries should actively review measures to strengthen tobacco control policies, taking the case of South Korea as an example. This can include expanding the definition of tobacco to encompass e-cigarettes and HTPs, and imposing restrictions on the advertising and promotion of e-cigarette products. Specifically, e-cigarette retailer websites often make baseless health claims and target young individuals through their marketing activities. The current marketing practices, which position e-cigarettes and HTPs as healthier alternatives to traditional cigarettes, encourage their use, and emphasize the latest trends, are not desirable considering the ongoing debate on the harmfulness of e-cigarettes and HTPs. Until the issues surrounding the harm of e-cigarettes and HTPs are resolved^20,21^, it is essential to monitor and prohibit the aggressive marketing practices of e-cigarette and HTP retailers. In particular, promotion strategies that contain exaggerated or evidence-free content about e-cigarettes and HTPs should be regularly monitored, measures such as suspension of sales should be considered, and strong legal punishment should be considered for retailers who sell e-cigarettes and HTPs to adolescents. Proactive regulation of these marketing strategies is urgently needed to prevent potential health risks associated with smoking in the future.

## Data Availability

The data supporting this research are available from the authors on reasonable request.
